# Proteome modifications on tomato under extreme high light induced-stress

**DOI:** 10.1186/s12953-018-0148-2

**Published:** 2018-11-24

**Authors:** Débora Parrine, Bo-Sen Wu, Bilal Muhammad, Keith Rivera, Darryl Pappin, Xin Zhao, Mark Lefsrud

**Affiliations:** 1 Department of Bioresource Engineering, Macdonald Campus, McGill University, 21,111 Lakeshore Boulevard, Sainte-Anne-de-Bellevue, Quebec H9X 3V9 Canada; 2 Department of Animal Science, Macdonald Campus, McGill University, 21,111 Lakeshore Boulevard, Sainte-Anne-de-Bellevue, QC H9X 3V9 Canada; 3 Cold Spring Harbor Laboratory, Cold Spring Harbor, NY USA

**Keywords:** LED, Psb28, PsbS, Wavelength, Photoinhibition, Salicylic acid, Chlorosis

## Abstract

**Background:**

Abiotic stress reduces photosynthetic yield and plant growth, negatively impacting global crop production and is a major constraint faced by agriculture. However, the knowledge on the impact on plants under extremely high irradiance is limited. We present the first in-depth proteomics analysis of plants treated with a method developed by our research group to generate a light gradient using an extremely intense light.

**Methods:**

The method consists of utilizing light emitting diodes (LED) to create a single spot at 24,000 μmol m^− 2^ s^− 1^ irradiance, generating three light stress levels. A light map and temperature profile were obtained during the light experiment. The proteins expressed in the treated tomato (*Solanum lycopersicum*, Heinz H1706) leaves were harvested 10 days after the treatment, allowing for the detection of proteins involved in a long-term recovery. A multiplex labeled proteomics method (iTRAQ) was analyzed by LC-MS/MS.

**Results:**

A total of 3994 proteins were identified at 1% false discovery rate and matched additional quality filters. Hierarchical clustering analysis resulted in four types of patterns related to the protein expression, with one being directly linked to the increased LED irradiation. A total of 37 proteins were found unique to the least damaged leaf zone, while the medium damaged zone had 372 proteins, and the severely damaged presented unique 1003 proteins. Oxygen evolving complex and PSII complex proteins (PsbH, PsbS, PsbR and Psb28) were found to be abundant in the most damaged leaf zone. This leaf zone presented a protein involved in the salicylic acid response, while it was not abundant in the other leaf zones. The mRNA level of PsbR was significantly lower (1-fold) compared the control in the most damaged zone of the leaf, while Psb28 and PsbH were lower (1-fold) in the less damaged leaf zones. PsbS mRNA abundance in all leaf zones tested presented no statistically significant change from the control.

**Conclusions:**

We present the first characterization of the proteome changes caused by an extreme level of high-light intensity (24,000 μmol m^− 2^ s^− 1^). The proteomics results show the presence of specific defense responses to each level of light intensity, with a possible involvement of proteins PsbH, Psb28, PsbR, and PsbS.

**Electronic supplementary material:**

The online version of this article (10.1186/s12953-018-0148-2) contains supplementary material, which is available to authorized users.

## Background

Plants cope with abiotic stress by either avoiding it or acclimating to it. Avoidance is the survival of the plant during unfavorable conditions as mature seeds, while acclimation to stress results in the modification of plant metabolism, which causes significant changes at the protein and gene-expression level [1, 2]. Both mechanisms reduce plant growth and yield, causing a major constraint to agriculture by negatively impacting global crop production [3, 4].

Although functional photosynthetic systems are required for plant survival, most species, when exposed to full sunlight, utilize as little as 10% of the absorbed light in the photosynthetic electron transport [5]. When excess energy cannot be dealt with, conditions of high light stress may cause irreversible photosystem damage, resulting in photoinhibition and the decrease of photosynthetic quantum yield [6, 7]. There are different strategies of photoprotection of the photosynthetic apparatus to control the excess exciting energy [8]. A well known mechanism is  the de novo synthesis and repair of proteins (as the D1 protein) that are essential to maintain photosynthesis [9, 10]. The D1 protein turnover is believed to have a protective role, by avoiding damage on PSI [11]. The oxygen-evolving complex is also damaged during high-light stress and it is the earliest signal of photoinhibition [12]. These mechanisms have been observed in commonly studied high-light stress conditions that are often up to 1000 μmol m^− 2^ s^− 1^ [13–15]. Here we investigate the changes in the tomato proteome resulted from an extreme light-induced stress of approximately 20,000 μmol m^− 2^ s^− 1^.

In this study, we performed the first characterization of the proteome of plants treated with an extremely high-light intensity (24,000 μmol m^− 2^ s^− 1^). To obtain a deep characterization of the light treatment impact, we applied a quantitative proteomics method (iTRAQ). The quantitative proteomics analysis determined the landscape of key proteins, and quantified the changes on protein expression. A high-intensity monochromatic light emitting diodes (LED) was used to create a light-induced stress condition on tomato leaves. This approach simultaneously generated three different zones of impact on the leaves at an extremely high light intensity (24,000 μmol m^− 2^ s^− 1^), a moderate intensity (~ 14,000 μmol m^− 2^ s^− 1^) and a low intensity (< 5000 μmol m^− 2^ s^− 1^).

The proteomics analysis revealed four different protein expression patterns, generating lists of similar expression behavior protein groups. The leaf area with the higher light impact exclusively presented proteins involved in the salicylic acid hormone synthesis. We also report on the high abundance and mRNA expression of proteins PsbH, PsbS, Psb28 and PsbR in comparison to other components of PSII and OEC in the most damaged leaf zone.

## Methods

### Plant growth and sampling

Tomato (*Solanum lycopersicum*) variety Heinz 1706 was provided by HeinzSeed Stockton, CA, USA. Heinz 1706 is the variety that was recently genetically sequenced [16].The tomato seeds were planted and grown hydroponically in rockwool (Grodan A/S, Dk-2640, Hedehusene, Denmark) and incubated under cool-white fluorescent bulbs (4200 K, F72T8CW, Osram, USA) in a growth chamber (TC30, Conviron, MB, Canada). The environmental conditions in the chamber were controlled at 50% relative humidity (RH), 25 °C light/dark temperature, an average of 390 ppm CO_2_, and a 16 h photoperiod with an irradiance level of 55 W m^− 2^ (approximately 250 μmol m^− 2^ s^− 1^). Fresh Hoagland nutrient solution was provided every other day. Hoagland composition [17]: 6.5 mM KNO_3_, 4.0 mM Ca(NO_3_)_2_.4H_2_O, 2 mM NH_4_H_2_PO_4_, 2.0 mM MgSO_4_.7H_2_O, 4.6 μM H_3_BO_3_, 0.5 μM MnCl_2_.4H_2_0, 0.2 μM ZnSO_4_.7H_2_0, 0.1 μM (NH_4_)_6_Mo_7_O_24_.4H_2_O, 0.2 μM CuSO_4_.5H_2_0, 45 μM FeCl_3_. Ten tomato plants were subjected to red LED light with an average irradiance level of 24,000 μmol m^− 2^ s^− 1^ on a ~ 1.1 cm^2^ spot in the center of a mature leaf for 5 min. After the light treatment, plants were kept in the growth chamber for a 10 day to recover and the observation of a bleached leaf area formation before tissue extraction. The leaves of each treatment of 10 plants were collected as one biological sample, to eliminate individual variances. The leaves were dissected and the areas corresponding to the light treated zone (Burned), adjacent (Limit) and rest of the leave (Regular) were kept separated, the remaining parts were discarded. Plant tissues were kept under − 80 °C before protein extraction. The control plant group was kept in the growth chamber during the full experiment without the intense irradiation and the experiment was replicated three times.

### Light treatment

A deep-red LED light (655 nm, LXML-PL01–0040, Philips-Lumileds, CA, USA) was used. The tomato leaves were placed 2.5 cm below the lights, where light intensity was at approximately ~ 24,000 μmol m^− 2^ s^− 1^, measured by a spectroradiometer (PS-300, Apogee, Logan, UT, USA). LED set up was as described by Wu et al. (not published). Briefly, the LED array was mounted to a water jacket connected to a water bath (ST-011, Guangzhou Rantion Trading Co., China) and a cluster concentrator optic (25 mm focal length, No. 263, Polymer Optics, Wokingham, Berkshire, UK) was placed in-front of the array. A focal spot of 12 mm diameter was generated by the cluster concentrator optic. An isotemp (4100R20, Fisher Scientific, Hampton, NH, USA) bath circulator was used to maintain a 0 °C coolant water bath. Filtered lenses were used to attenuate the light, to measure the high irradiance level with the use of the spectroradiometer. Leaf temperature was measured in three biological replicates as reported by [18] with a copper constantan thermocouples (type T, 0.03 mm, Omega Engineering Canada, QC, CA). The temperature was measured during the 5 min before and after the light treatment as well as during the 5-min treatment. The thermocouples were placed on the surface of the leaf using glue extracted in chloroform from adhesive tape, data points were collected every second.

### Tissue lysis, protein extraction and tryptic digestion

Fresh plant tissue (20 mg) was treated with 500 μL of lysis buffer (5% SDS, 50 mM triethylammonium bicarbonate buffer (TEAB)). A volume of 5 μl of each of protease inhibitor cocktail 1, phosphatase inhibitor cocktail 2, and phosphatase inhibitor cocktail 3 (Sigma-Aldrich, MO, USA) were added to the sample. The samples were then mixed at 1250 rpm for 30 min. Tris(2-carboxyethyl)phosphine (TCEP) was added to 150 μL of the lysate to a final concentration of 5 mM. Samples were heated to 55 °C for 20 min, allowed to cool to room temperature, and methyl methanethiosulfonate (MMTS) was added to a final concentration of 10 mM. Samples were incubated at room temperature for 20 min to complete blocking of free sulfhydryl groups. Methanol was added at 4x the sample volume to precipitate proteins, chloroform was added at 2x the sample volume, and deionized water was added at 3x the sample volume. The samples were then incubated at − 20 °C for 2 h and centrifuged at 5000 rpm for 10 min at 4 °C. Methanol was added at 3x the original sample volume and the sample was vortexed. The sample was centrifuged at 14,000 rpm for 10 min at 4 °C. The proteins were reconstituted with 60 μL of lysis buffer and a BCA assay (Pierce, Thermo Fischer Scientific, MA, USA) was performed to determine protein concentration. Proteins were digested by applying 80 μg of each lysate to S-Trap™ mini spin columns (ProtiFi, NY, USA) according to the manufacturer instructions. Briefly, lysates were acidified with phosphoric acid to a final concentration of 1.2% and added to an S-Trap™ containing 6x lysate volume of s-trapping buffer (90% Methanol, 100 mM TEAB). Digestion was carried out with 2 μg of sequencing grade trypsin (Promega, WI, USA) in 125 μL of 50 mM TEAB and was added to the S-Trap™ which was incubated overnight at 37 °C. The peptides were eluted from the column with subsequent applications of 50 mM TEAB, 0.2% formic acid in water and 0.2% formic acid in 50% acetonitrile. After dried in a vacuum, peptides were then reconstituted in 50 μL of 0.5 M TEAB/70% isopropanol and labeled with 8-plex iTRAQ reagent for 2 h at room temperature, according to [19]. The eight labeled samples were then acidified to pH 4 with formic acid, combined, and concentrated in vacuum until ~ 10 μL remained.

### iTRAQ-labeling and LC-MS/MS

An Orbitrap Fusion Lumos mass spectrometer (Thermo Scientific, MA, USA), equipped with a nano-ion spray source was coupled to an EASY-nLC 1200 system (Thermo Scientific, MA, USA). The LC system was configured with a self-pack PicoFrit™ 75 μm analytical column with an 8 μm emitter (New Objective, Woburn, MA) packed to 25 cm with ReproSil-Pur C18-AQ, 1.9 μM material (Dr. Maish GmbH, Ammerbuch-Entringen, DE). Mobile phase A consisted of 2% acetonitrile 0.1% formic acid and mobile phase B consisted of 90% acetonitrile 0.1% formic acid. Peptides were then separated using the following steps: at a flow rate of 200 nL/min: 2% B to 6% B over 1 min, 6% B to 30% B over 84 min, 30% B to 60% B over 9 min, 60% B to 90% B over 1 min, held at 90% B for 5 min, 90% B to 50% B over 1 min and then flow rate was increased to 500 nL/min as 50% B was held for 9 min. Eluted peptides were directly electrosprayed into the Fusion Lumos mass spectrometer with the application of a distal 2.3 kV spray voltage and a capillary temperature of 300 °C. Full-scan mass spectrum (Res = 60,000,400–1600 m/z) was followed by MS/MS using the “Top N” method for selection. High-energy collisional dissociation (HCD) was used with the normalized collision energy set to 35 for fragmentation, the isolation width set to 1.2 and a duration of 10 s for the dynamic exclusion with an exclusion mass width of 10 ppm. Monoisotopic precursor selection was used for charge states 2+ and greater, and data were acquired in profile mode. Each biological replicate consisted of samples collected from 10 treated or control plants, to account for biological variability, two biological replicates for each treatment and control were differently labeled and analyzed by LC-MS/MS. An empirical distribution representing total experimental variability was built, not just within each group. This was done by comparing the ratios of all replicates within each condition and forming an empirical Cumulative Distribution Function (CDF). The CDF contained the ratio of every replicate regardless of condition for all proteins identified which represented both the biological and technical variability of this dataset. The fold change cutoff for significance was determined by selecting only ratios values more than 2 standard deviations from the mean. In this study, 90% of ratios between the replicates fell between 0.61 and 1.61, with values outside this range being significant at a FDR adjusted *p*-value equal to 0.05. A minimum of two unique peptides per proteins was also included as a quality filter.

### Data analysis

Peaklist files were generated by Mascot Distiller (Matrix Science, MA, USA). Protein identification and quantification were carried using Mascot 2.4 against the *Solanum lycopersicum* cv. Heinz 1706 database (UniProt, proteome reference: UP000004994). Methylthiolation of cysteine and N-terminal, and lysine iTRAQ modifications were set as fixed modifications, methionine oxidation and deamidation as variable. Trypsin was used as cleavage enzyme with one missed cleavage allowed. Mass tolerance was set at 30 ppm for intact peptide mass and 0.3 Da for fragment ions. Search results were rescored to give a final 1% false discovery rate (FDR) using a randomized version of the same tomato database. Protein-level iTRAQ ratios were calculated as intensity weighted, using only peptides with expectation values < 0.05. Global ratio normalization (summed) was applied across all iTRAQ channels. Protein enrichment was then calculated by dividing sample protein ratios by the corresponding control sample channel.

### Bioinformatics analysis

Functional annotations of the identified proteins were obtained via the UniProt Gene ontology tool (UniProt-GOA) [20]. Protein interaction network was predicted by the STRING database [21], which obtains interactions based on genomic, experimental, co-expression or previous knowledge information context at the function or physical level. The analysis was performed under the highest confidence (0.9) interaction score. Pathway annotation was obtained by the Kyoto Encyclopedia of Genes and Genomes (KEGG) database. The KEGG Pathway Annotation tool generated a visualization of the pathways involved in the high abundant proteins of the high light treated samples and control. A hierarchical clustering analysis was applied to clustering proteins based on their Euclidean distance and complete linkage to visualize the different trends of protein abundance of the dataset. The log2 transformed protein ratios (treatment/control) were clustered by the hierarchical clustering function of the Perseus software.

### RT-qPCR

Total RNA from leaf samples was extracted with the RNeasy® Plant Mini kit (Qiagen, Germany). QuantiTect® Reverse Transcription kit (Qiagen) was used to synthesize the cDNA as presented in the manufacturer’s protocol. RT-qPCR primers were designed using the online tool Primer-BLAST from the National Center for Biotechnology Information (NCBI) (Table 1). The mixed solution of RT-qPCR reaction contained Platinum® SYBR® Green qPCR SuperMix-UDG with ROX (2×, Invitrogen, USA), reverse and forward primers mix (4.28 μM) and 20-fold-diluted cDNA template. All reactions were performed on a CFX Connect Real-Time PCR system (Biorad, USA). Reaction conditions were 10 min at 95 °C, followed by 40 cycles of heating at 95 °C and annealing at 60 °C for 15 and 60 s, respectively. Melting curves were carried out in each RT-qPCR to verify single-product amplification. The relative level of gene expression was calculated with the Livak method (2- (ΔΔCt)). The genes protein phosphatase 2A catalytic subunit (*PP2Acs*) [22] and clathrin adaptor complex subunit (*clat*) [23] were used as the reference genes. Measurements were recorded from three technical and three biological replicates for each experimental condition.

**Table 1:** DNA primers for RT-qPCR used in this study

Gene	Forward primer	Reverse primer	PCR product size (bp)	Source
*psb28*	CCTCGCTCTCTTCTCGGAAT	GCAAAACGCGAACGGGATAG	98	This study
*psbS*	GGAATTGGCTTCACTAAGCA	AGTGGCTCTGCTTCATAGAT	155	This study
*psbH*	TCTGGTCCAAGACGAACTGC	CAAAGGGGTAGTTCCCCACC	93	This study
*psbR*	CAGGAAGCCCAAGGGAAAGG	GTCACCGCCCATATGGCTAA	153	This study
*TPP2Acs*	CGATGTGTGATCTCCTATGGTC	AAGCTGATGGGCTCTAGAAATC	149	Løvdal and Lillo, 2009
*clat*	ATGCAATCACACCAGCAC	ACTCAGCACAACAACAAAGG	61	Dekkers et al., 2012

### Statistical data analysis

All data reported are the average of replicates. A two-way variance analysis (ANOVA) was applied, and pair-wise comparisons were adjusted with Tukey’s test (*p* ≤ 0.05), unless otherwise indicated.

## Results

### High light treatment

**Fig. 1:**
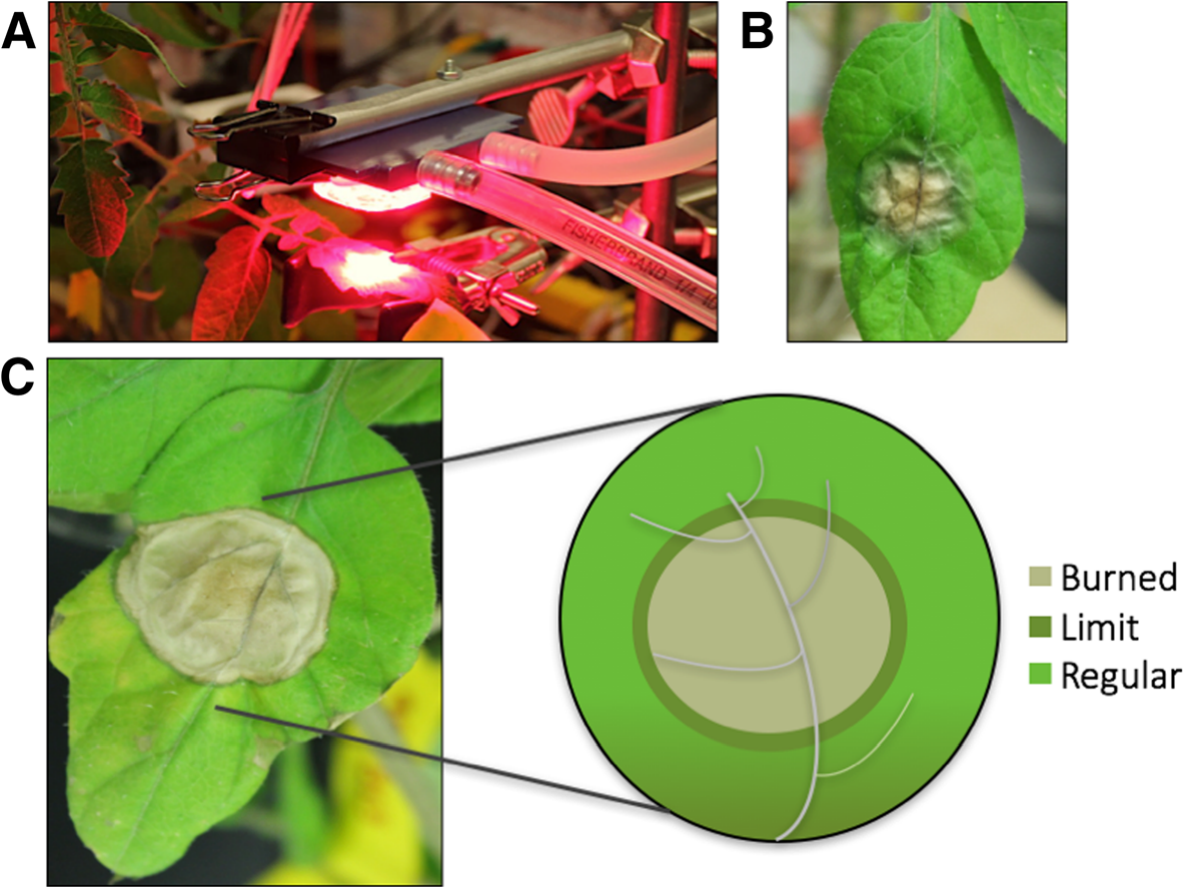
LED light treatment schematic. (**a**) Light treatment on tomato leaves using high-intensity deep-red (655 nm) LED light at 24,000 μmol m^− 2^ s^− 1^ (**b**) Tomato leaf after high-intensity light treatment (**c**) Photo of the leaf after the 10-days period after the high light treatment and sampling zone scheme: Burned, Limit, and Regular

In this study, a new methodology of light treatment was utilized to generate extreme heat on a spot of a plant leaf. A deep-red LED (655 nm) was chosen due to the well-characterized plant physiological response to red light [24]. The light treatment generated a visible highly dehydrated area on the tomato leaves (Burned area), corresponding to the area treated with the highest light intensity. The light damaged leaf zones were better defined after a 10-days period, when a dark line was visible between Burned and Regular, which we defined as the Limit sample (Fig. 1). The control plants were grown for the whole period of the experiment (40 days) under normal light conditions (250 μmol m^− 2^ s^− 1^), and its protein abundance was used for comparison amongst the treated samples.

**Fig. 2:**
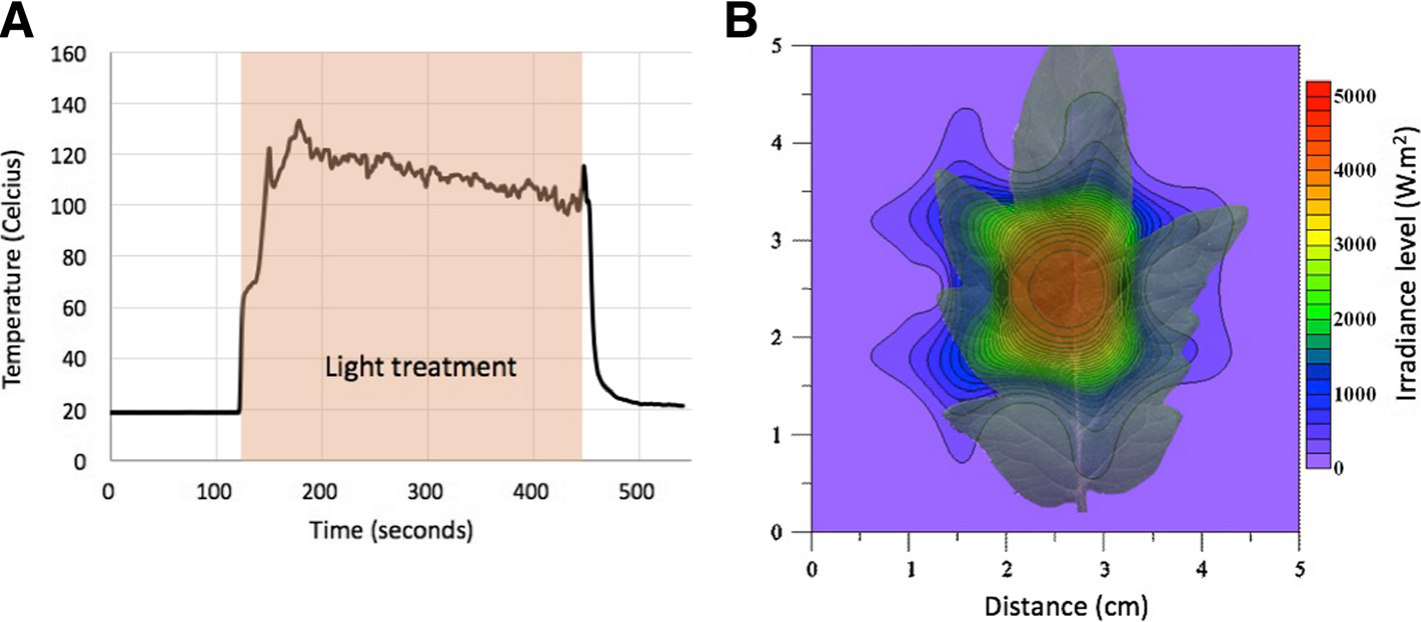
Light treatment temperature and light measurements. (**a**) Leaf temperature measurement by thermocouple (0.03 mm, type T, Omega Engineering Canada). Data points collected every second before, during and after light treatment. The red arrow indicates the duration of the light treatment (~ 5 min). (**b**) Light mapping with a projected tomato leaf showing the irradiance distribution on the tomato leaf generated by the high-intensity light treatment with the red LED. The inner circle shows the area corresponding to the Burned sample. The average of three biological replicates is reported

The use of LEDs instead of conventional lights for plant experimentation allows the generation of narrowed width wavelength, increasing the color specificity. More importantly, LEDs has non, or low, heat emission, and are considered low-temperature lamps [25]. Further, using a concentrator optic, we generated an extreme high irradiance level of 24,000 μmol m^− 2^ s^− 1^in a well-defined leaf spot (Burned). To determine the light intensity distribution on the leaf, a light map was generated and can be seen in Fig. 2b. Although the Burned spot had the maximum light intensity of 24,000 μmol m^− 2^ s^− 1^, the other zones also presented high-intensity levels, the Limit zone intensity was of ~ 14,000 μmol m^− 2^ s^− 1^, and the Regular measured < 5000 μmol m^− 2^ s^− 1^. Because LEDs are low-temperature lights, we did not expect a high increase in leaf temperature caused by the LED apparatus, but rather by the plant. Heat dissipation is a normal response of plants to high light, and it is known as the non-photochemical quenching mechanism. This phenomenon is a result of the excess of energy absorbed by the plant that is released as heat. In this response, the singlet excited state chlorophylls, returns to ground state by heat dissipation, or fluorescence emission, since not all the energy can be directed to photosynthesis reactions [26]. To detect the temperature change, a thermocouple was used to monitor the tomato leaf during the light treatment (Fig. 2 a). The highest temperature measured was detected after 75 min after the beginning of the treatment and corresponded to ~ 138 °C.

### Protein identification and functional enrichment analysis

#### Functions of proteins found in the zones under lower irradiance (limit and regular)

**Fig. 3:**
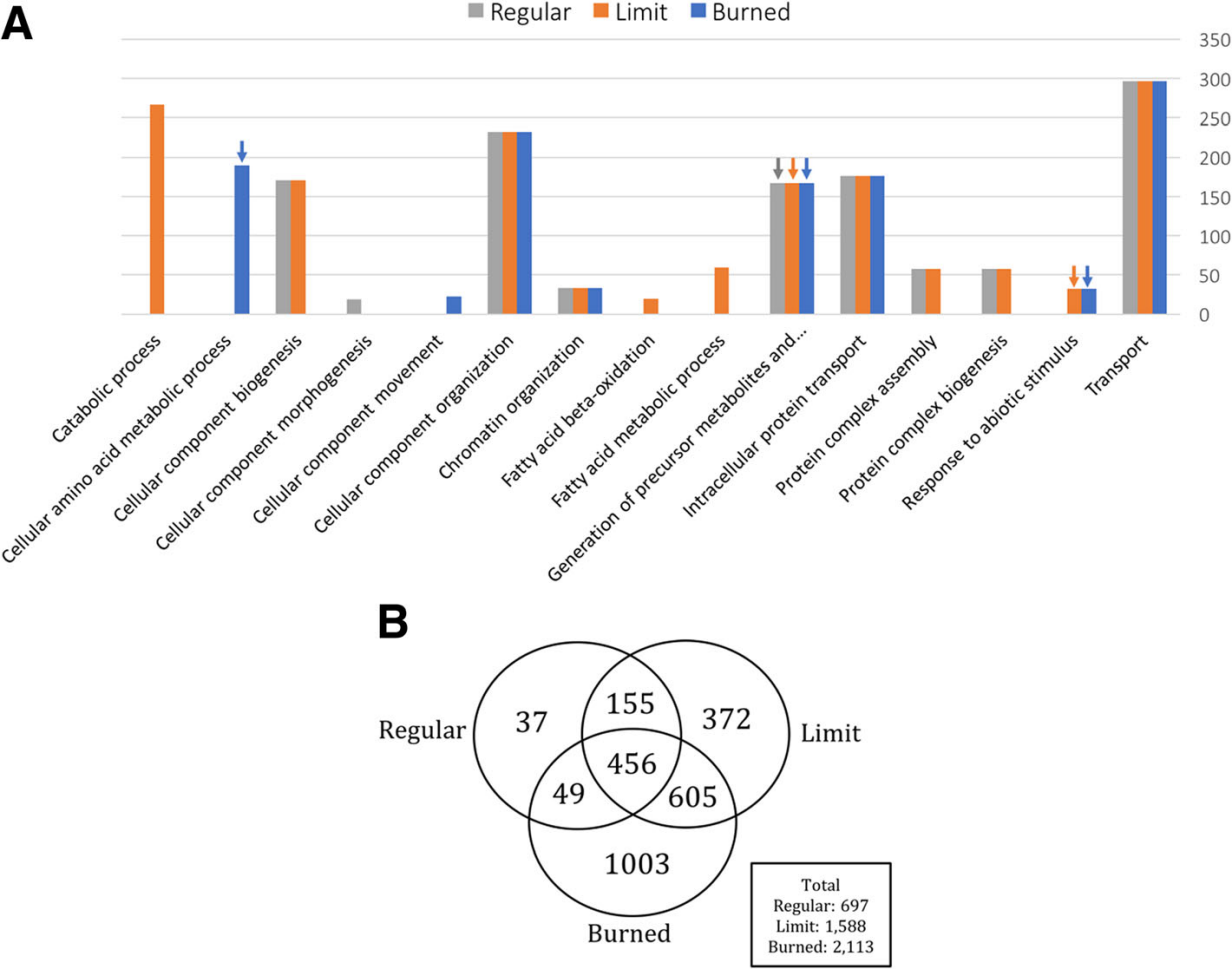
Quantitative functional enrichment of the detected proteins expression ratios and Venn diagram of differentially abundant proteins. **a** Statistically differentially abundant proteins (*p* < 0.05) from tomato leaves recovered from light-induced heat stress (Burned, Limit, and Regular) grouped in a Venn diagram. **b** Proteins present in samples Burned, Limit, and Regular were assigned GO-slim sub-categories of the biological processes category by the PANTHER enrichment test. Bonferroni correction was applied for multiple testing. Of the total 3994 proteins, 3873 were mapped to a sub-category, the expression ratios were used to weight the representation of the sub-category regarding the overall expression, y-axis show number of proteins. Only significant matches (p < 0.05, Wilcoxon Rank-Sum test) top 15 categories are shown. The arrows indicate that the protein expression is shifted towards smaller values than the overall expressions. Notable trends are the high proportion of protein expression related to the cellular process in the Regular sample, when compared to Burned and Limit samples

We identified 5577 proteins in 1% FDR, which were further filtered to only proteins containing two or more unique peptides. The filtered list resulted in 3994 proteins that were further analyzed. In order to identify the protein functional groups, found in each light treated sample, the protein expression ratios (treatment/control) were calculated and used as input in a functional enrichment test. From 3994 proteins, 120 proteins did not present functional annotation and could not be mapped, one protein presented multiple mapping information. The PANTHER enrichment test [27] (v. 12.0) was performed to obtain the proportion of each GO-slim category in relation to the protein expression of each sample treatment (Fig. 3a).

In the Regular sample, the trends of sub-categories with unique proportion of the overall expression values were: cellular process, RNA metabolic processes, and purine nuclease metabolic process. The Limit sample unique proportion of proteins were in carbohydrate transport, fatty acid metabolic process, and fatty acid beta-oxidation.

#### Functions of proteins found in the zones under higher irradiance (burned)

The Burned sample had unique overrepresented categories as cellular amino acid biosynthetic process, glycogen metabolic process, glycolysis, mRNA processing, mRNA splicing via spliceosome, protein localization, RNA splicing via transesterification reactions, steroid metabolic processes, and transcription DNA dependent. Although a high number of proteins were found to be part of the carbohydrate metabolic process, cellular amino acid metabolic process, metabolic process, monosaccharide metabolic process, generation of precursor metabolites, and energy, the expression values of these categories were sub-represented in the overall protein expression of the sample.

The functional analysis showed a higher diversity of upregulated functions in the Burned sample, when compared to the less impacted zones (Limit and Regular), also due to the presence of a high number of upregulated proteins. A general upregulation of RNA synthesis and processing activity shows a general increase in transcription in the Burned sample.

### Expression patterns trends from differentially expressed proteins

We calculated an abundance ratio threshold of > 1.61 and < 0.61 based on the biological replicates variability and applied it to obtain a list of statistically differentially abundant proteins (*p* < 0.05). Interestingly, when analyzing the proteins detected in all samples, the Burned sample had a high total of differentially abundant proteins (2113), while the Limit samples had 1588 proteins (Fig. 3b). The Regular sample had the lowest number of differentially abundant proteins (697), due to the lower light intensity that the sample was treated with, and, therefore, were similar to the proteins from the control sample. All three samples shared 456 differential proteins in common. The Burned sample presented the highest number of uniquely differential proteins (1003). To our knowledge, this is the first time the unique set of proteins from the Burned sample are reported as involved in the recovery of a highly damaging light intensity stress since this is the first proteomics study of the light treatment applied in this work.

**Fig. 4:**
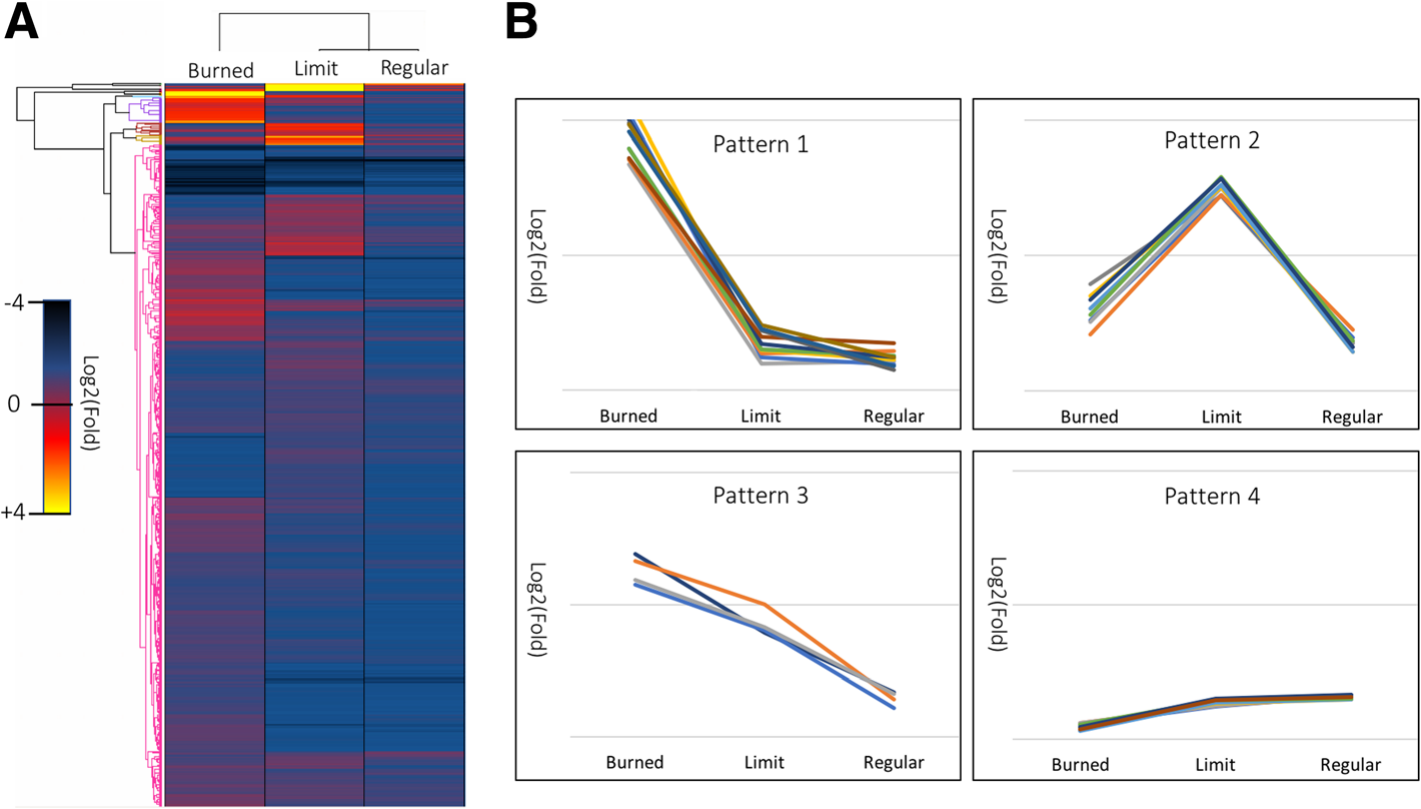
Hierarchical clustering analysis of differentially abundant proteins and cluster expression patterns. **a** Differentially regulated proteins (*n* = 2680) in Control, Burned, Limit and Regular were clustered into groups with similar log2 transformed ratio (treatment/control) patterns. The Euclidian distance was chosen to cluster proteins by abundance traits. The 8 protein clusters presenting differential abundance trends were chosen for further analysis. **b** The graphical representation of the four protein abundance patterns (Pattern 1–4) found in the clusters obtained by the hierarchical clustering analysis (sample in x-axis, fold-change on y-axis). Clusters were assigned to groups with a similar pattern and for each pattern, a list of proteins was generated. 112 proteins were part of pattern 1, 102 proteins were part of pattern 2, pattern 3 had 13 proteins and pattern 4, the largest group, had 2453 proteins

A Hierarchical cluster analysis was carried out to investigate the light-induced stress recovery of tomato plants. The analysis was applied to the subset of differentially abundant proteins of samples Burned, Limit, and Regular (*p* < 0.05, greater than a 1.61-fold abundance difference) (Fig. 4a). The protein expression values used as an input for the analysis are the normalized ratios of the treatment and control (Burned/Control, Regular/Control, and Limit/Control). A total of 14 clusters of proteins with similar expression trends, with either a higher expression seen in the Burned, or Limit samples, or a linear relation to light intensity, or a similar expression in all samples, were defined. From the 14 clusters, we observed four patterns of different relative abundance (a description of the four patterns is presented in Fig. 4b) (see Additional files 1 and 2 for a list of proteins per cluster). Protein lists were generated for each of the four different expression patterns, a total of 112, 102, 13, and 2453 proteins were represented in patterns 1–4, respectively. The protein groups of each of the patterns were analyzed for their function through analysis of their associated GO term in the Biological Processes category and are resumed in Table 2.

**Table 2:** Main functions of proteins found in pattern 1 to 4 from the hierarchical clustering analysis of the ratio of differentially abundant proteins found in the Burned, Regular, and Limit samples in relation to the control samples (*n* = 2680 proteins)

Pattern	Pattern 1	Pattern 2	Pattern 3	Pattern 4
Number of proteins	112	102	13	2453
Functions	Photosynthesis II reaction center, peroxidases, fruit-ripening protein.	Non-specific lipid-transfer proteins, pathogenesis related proteins, pathogenesis-related proteins.	Carbohydrate binding, catalytic activity, nucleic acid binding.	Anatomical structure, biosynthetic processes, cellular processes, carbohydrate metabolic processes, response/defense functions, chlorophyll a-b binding proteins, photosystem I iron-sulfur center, components of photosystem II, proteins D1, D2, CP47, CP43.

Proteins with the pattern 1 expression behavior presented high abundance in the Burned sample, and similar lower expressions in Limit and Regular samples. The trend of pattern 1 may indicate proteins with a role in long-term recovering plant tissues from extreme light damage, as they presented a very high expression value in the Burned sample. Photosystem II reaction center protein, two peroxidases, and fruit-ripening protein, were some of the proteins found in this group.

Proteins with pattern 2 showed higher expression values in the Limit sample and lower similar values for Burned and Regular. This group presented four Non-specific lipid-transfer proteins, three Pathogenesis-related proteins, which are proteins part of the general response/defense response to stimulus function.

The group of proteins represented by pattern 3 had high expression values in the Burned sample and decreasing values from the Limit to the Regular samples, following the intensity trend of the light treatment (high on Burned, lower on Limit, and low on Regular). The most frequent function was the role in binding (carbohydrate binding, catalytic activity, nucleic acid binding) and defense response. This group of proteins was characterized by mostly DNA/RNA regulators, and proteins related to defense response. Furthermore, pattern 3 group is interesting because the proteins in this group can be explored by scientists interested in light regulated genes.

The expression values of pattern 4, the largest group were either similar in all the three samples or slightly higher in the Regular sample. The proteins assign to this group were related to a large variety of roles as anatomical structure, biosynthetic processes, cellular processes, and carbohydrate metabolic processes. 37 proteins had response/defense functions, of a total of 2453 proteins. Interestingly, this group contained 12 chlorophyll a-b binding proteins (LHCB), photosystem I iron-sulfur center, eight components of photosystem II, and proteins D1, D2, CP47, CP43, which are found to be involved in light stress conditions, and particularly, repair of photosystem II from photodamage, showing that, even though the tomato plants recovered for a period of 10 days, these proteins were still found from the high light stress [7, 28], and the photosystem damage repair mechanisms could still be detected.

### Network of differentially expressed proteins

#### Network of proteins found in the leaf zones under higher irradiance (burned)

We analyzed the differentially abundant proteins from each sample to visualize pathways that are involved in the impact of different levels of light damage on the plants leaves. The set of differentially abundant proteins unique to the Burned sample (1003 proteins) showed an enrichment of antennas from photosystem I LHCA1, LHCA2, LHCA4 and from photosystem II LHCB1, LHCB3, LHCB6, which are implicated in photoprotection mechanisms [29–31]. While most components of the PSII complex and OEC appeared to be expressed at the same level as in the control sample, four of them were enriched: PsbH, PsbS, Psb28 and PsbR.

**Fig. 5:**
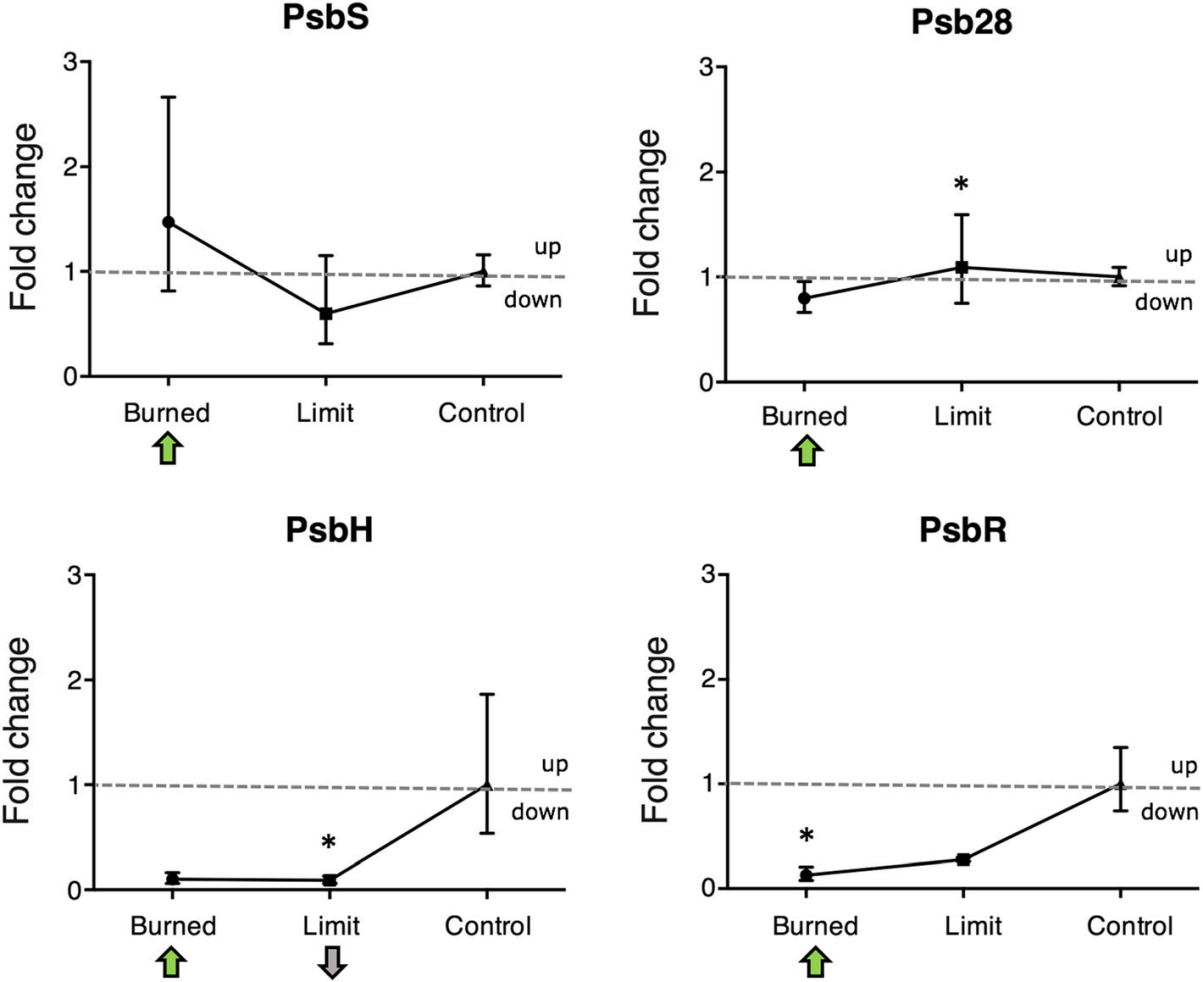
Comparison of transcription and translation levels of genes of interest. mRNA fold changes of PsbH, PsbR, Psb28, and PsbS in the Limit, Burned and control detected by RT-qPCR. Controls used were *clat* and *PP2Acs*. The data were analyzed by ANOVA and was adjusted for multiple comparison of the means with a Tukey test at *p* = 0.05 significance level. Statistically significant differences from the control are indicated by *. Error bars show standard deviations with *n* = 6 replicates. Arrows indicate the direction of the significant protein fold (green, high abundance, grey, low abundance), the absence of arrows indicates no change in the protein abundance between sample and control

An analysis of the mRNA expression of the four proteins was carried out to study the correlation between the protein and the mRNA expression (Fig. 5). PsbS mRNA levels shows a trend to be higher in the Burned sample, compared to the Limit and Regular (although not statistically significant). Psb28 showed similar levels to the control, and was slightly increased in the Limit sample. PsbH and PsbR had lower levels of expression when compared to the control. PsbS mRNA and protein levels agreed, being higher than the control. The Psb28 expression trend to be higher than the control was not followed by the mRNA, being similar to the control level. PsbH and PsbR did not follow the protein expression level, showing lower values than the control.

The light treatment in the Burned sample involved a general overexpression of the metabolism of proteins related to nitrogen compound metabolic process (such as fructose-bisphosphate aldolase, Glucose-6-phosphate dehydrogenase, glutamine synthetase), primary metabolism (carbohydrate, protein, and lipid processes), as ATP synthase, photosystem components, 50S ribosomal protein, and other primary metabolic functions, as methylation, developmental processes, growth, and reproductive process.

**Fig. 6:**
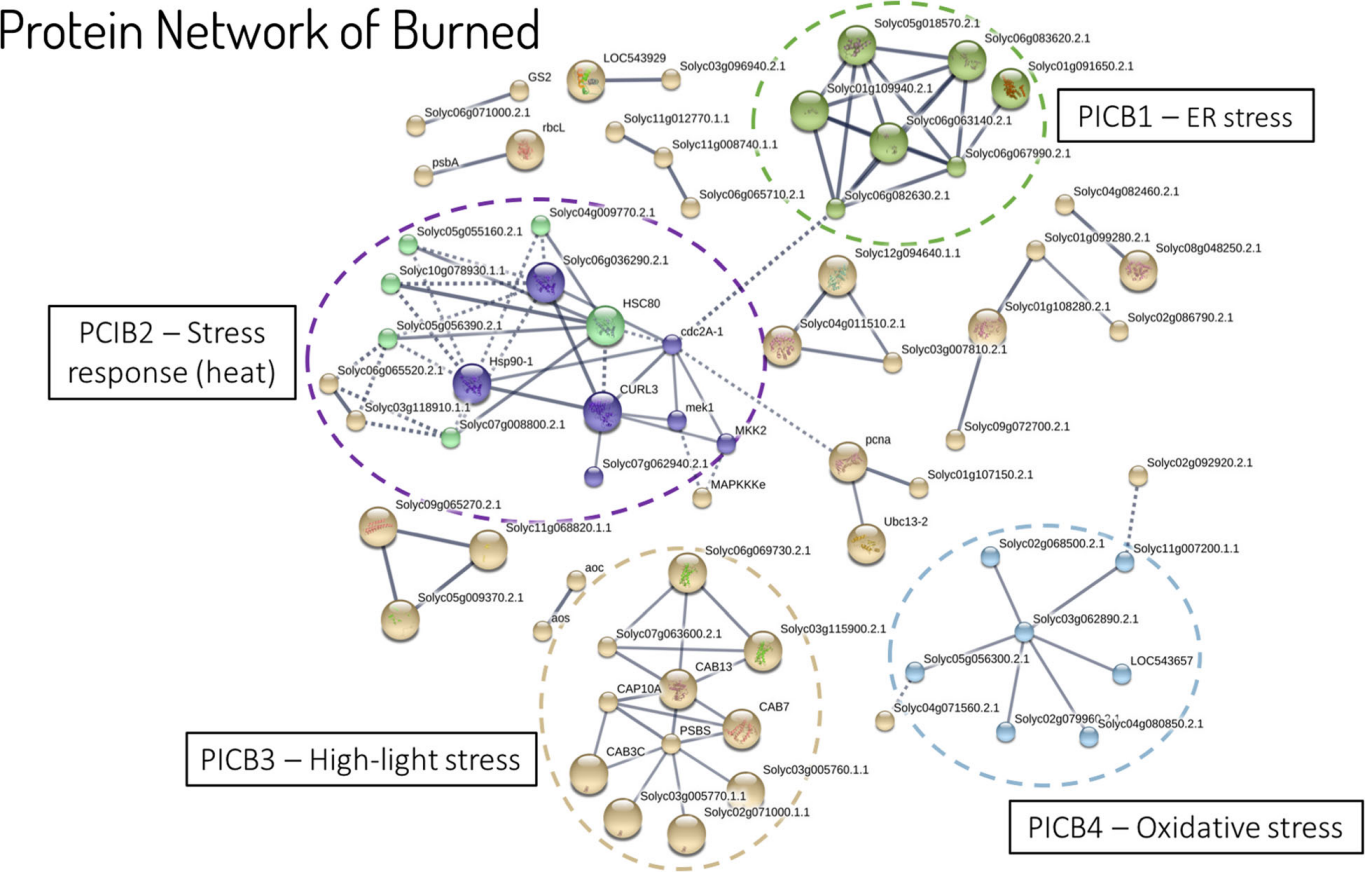
Protein interaction network from differentially abundant proteins found in the Burned sample. The STRING software was used to obtain a network of protein interactions at high confidence (0.7). Cluster analysis by K-means resulted in four well-defined clusters

Since the differentially expressed protein of the Burned sample formed a large dataset (1003 proteins), we used the functional analysis to filter the functions of interest. GO terms were assigned to the whole dataset and the 194 proteins from the enriched terms immune system process and response to stimulus were further investigated. In order to better understand the role of the uncharacterized proteins and their relation to the other proteins of the dataset, the filtered proteins were analyzed with STRING [32] to obtain protein-interaction networks. The generated network was analyzed applying high confidence (0.7) and K-means clustering. Four proteins interaction clusters (PICB) were defined in the Burned sample (Fig. 6). The protein interaction analysis revealed four well-defined clusters of interactors.

PICB1 had seven uncharacterized proteins with a role in the functional terms: positive regulation of RNA polymerase II transcriptional preinitiation complex assembly, ubiquitin-dependent ERAD pathway, DNA repair, protein K63-linked deubiquitination, and response to the absence of light. PICB2 presented proteins with functions: response to heat, response to stress, regulation of gene expression, stress-activated protein kinase signaling cascade, brassinosteroid mediated signaling pathway, response to unfolded protein, and chaperone-mediated protein folding. PICB 3 functions were related to response to light stimulus, and nonphotochemical quenching, where 10 of the 11 proteins where chlorophyll binding proteins. PICB 4 had functions as response to high light intensity, response to salt stress, negative regulation of plant-type hypersensitive response, and cellular response to oxidative stress.

#### Network of proteins found in the leaf zones under lower irradiance (limit and regular)

**Fig. 7:**
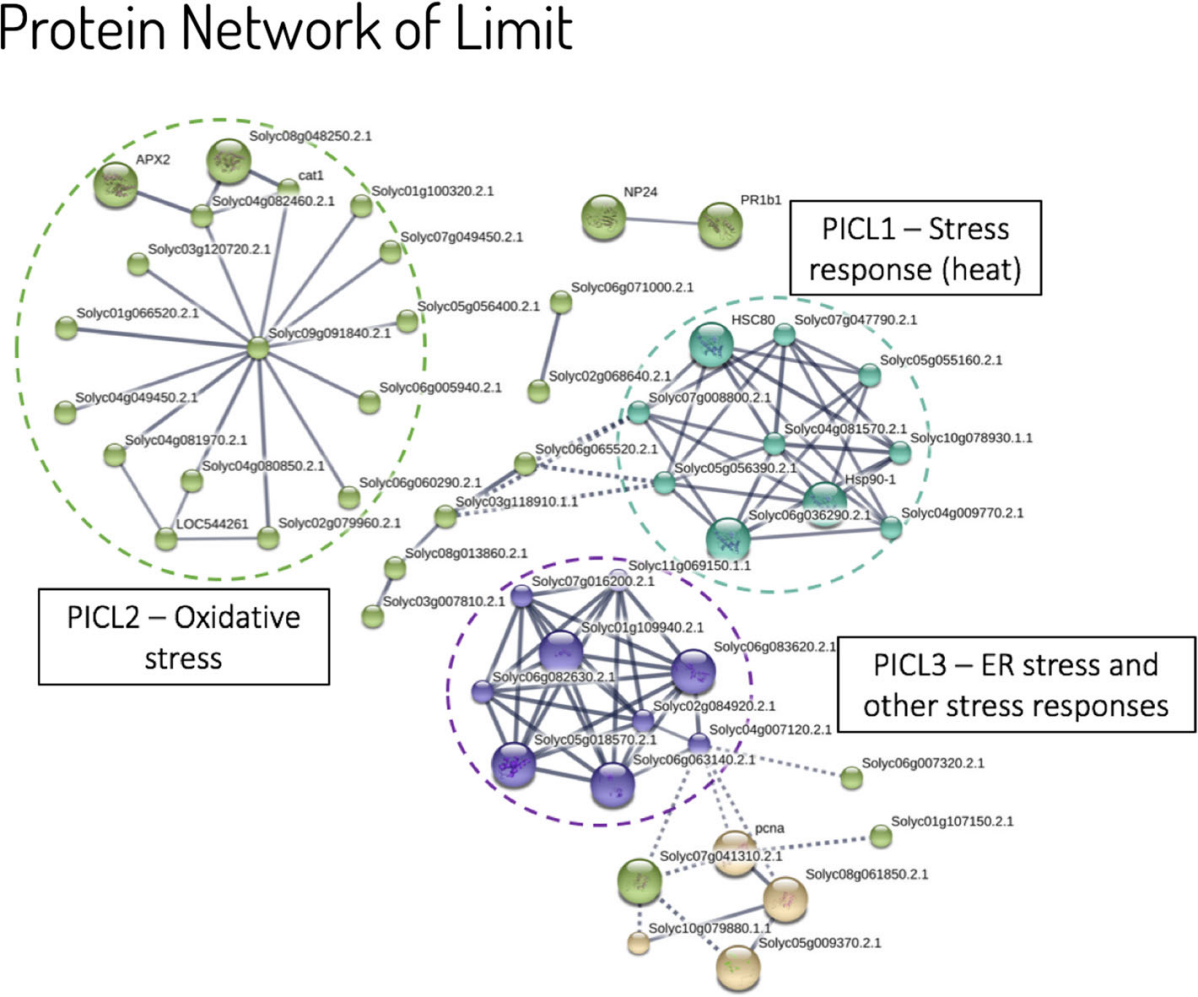
Protein interaction network from differentially abundant proteins found in the Limit sample. The STRING software was used to obtain a network of protein interactions at high confidence (0.7). Cluster analysis by K-means resulted in three well-defined clusters

The same analysis was carried out with the Limit sample to compare to the response of the Burned sample. A protein interaction network was obtained from the 155 proteins from immune system process and response to stress. Three clusters (PICL) were evidenced by the protein-interaction network analysis (Fig. 7). PICL 1 was composed of proteins related to chaperone, response to heat, response to cold, salt, and drought. PICL 2 proteins were related to response to light stimulus, response to hydrogen peroxide, response to endoplasmic reticulum stress, cellular response to oxidative stress, response to absence of light, response to oxygen radical. PICL 3 contained proteins with functions such as ubiquitin-dependent ERAD pathway, proteasome-mediated ubiquitin-dependent protein catabolic process, response to salt stress, and defense response to fungus, incompatible interaction.

The set of proteins from the Regular sample were also accessed for protein interactions. A total of 85 proteins were mapped. However, only 17 proteins formed interactions, the larger cluster being formed of 5 proteins related to heat stress and chaperones, followed by three clusters of three proteins each (the list of proteins is provided in the Additional files 1 and 2).

## Discussion

The proteomics analysis of the tomato leaves tissues under the high light treatment revealed 1003, 372, and 37 proteins specific to the samples with different light intensity (Burned, Limit, and Regular, respectively). We obtained three different levels of light-induced heat stress defense in the same leaf tissue, meaning three different highly localized stress defense responses. Through a clustering analysis, we identified proteins that respond to the increase of light intensity in a direct relationship (i.e., protein concentration augments with the increasing of light intensity) and proteins that are only abundant in the higher light intensity (~ 24,000 μmol m^− 2^ s^− 1^) or the medium (~ 14,000 μmol m^− 2^ s^− 1^).

Similar protein response in all the samples represented the largest group, as expected. In this group, a general enrichment of primary metabolic functions was seen. From a total of 2455 proteins, 212 were assigned to response to stimulus function, 8 to immune system process, three to removal of superoxide radical, and three to circadian rhythm. The response to stimulus proteins were mostly of proteins related to biotic stress (70 proteins) and chemicals (82 proteins). Since we were more interested in the uniqueness of the differentially abundant proteins in each sample and pattern 4 is composed of proteins upregulated in all samples, we decided to explore patterns 1–3.

### Active functions in response to medium-light intensity (limit and regular)

Proteins with higher expression in the Limit sample and following pattern 2 included non-specific lipid-transfer proteins, annexin proteins, R1 and PR10, and xyloglucan endotransglucosylase /hydrolases, with functions as Response to biotic and abiotic stresses, hormones, chemicals, and external stimulus. The function of the non-specific lipid-transfer proteins is still not well defined in the literature, they have been considered in the transport of monomer of cutin, deposition of lipophilic cuticular material, and plant defense [33]. More recently, it has been shown to have a positive impact on drought and low-temperature stresses, where the non-specific lipid-transfer protein transcript levels were decreased in response to salicylic acid and increased during methyl jasmonate treatment [33]. Attention has been focused on non-specific lipid-transfer proteins due to their role as major allergens, along with their enzymatic and heat resistance.

Annexins have been shown to be downregulated in response to low-light stress response in cotton (*Gossypium hirsutum* L.) [34]. In *Arabidopsis*, phytochrome-mediated changes in annexin expression have been studied, showing a high level of *AnnAt5* transcript response to red light stimuli [35]. Still, in *Arabidopsis*, the overexpression of annexin *AnnAt1* improved drought tolerance and mitigated Reactive oxygen species (ROS) response [36]. In tomato subjected to drought stress, the auxin interactor *SpUSP* increased expression of LHCB and activated other photosynthesis-related genes, maintaining regular photosynthesis levels by keeping the antenna integral while reducing ROS impact [37]*.* R1 protein is a regulator of starch degradation in plants, and R1 deficiency has generated reduced starch phosphorylation and high starch accumulation generating phenotype with starch excess in potato and *Arabidopsis* [38].

Recently, abscisic acid-induced leaf starch degradation has been reported to have an important role in osmotic stress regulation, having a synergistic role of enzymes regulated by abscisic acid through AREB/ABF-SNRK2 kinase-signaling pathway in an action to maintain carbon deviation to the roots and osmolyte accumulation [39, 40]. Pathogenesis-related proteins (PR) are reported to be involved in different stress defenses to biotic stresses and pathogens. The overexpression of PR10 has been shown to increase salt tolerance in transgenic *Arabidopsis* containing the *SmPR10* gene from *Salix matsudana* Koidz and salt and drought tolerance in rice [41, 42]. Xyloglucan endotransglucosylase hydrolases proteins regulate cell wall extension, construction and metabolism, are involved in the cell wall hemicellulose synthesis, and plant response to environmental stresses caused by heavy metal, salt, and drought [43].

### Active functions in response to high light intensity (burned)

Besides seeing a difference in the expression behavior, we used the protein-interaction network analysis to better visualize the different levels of light-induced stress responses represented in the Regular, Limit, and Burned samples. Presenting a higher level of complexity when compared to prokaryotic organisms, plant tissues are estimated to contain a pool of 10,000 proteins at any stage [44]. However, the sample preparation efficiency and mass spectrometry technology are limitations that highly impact the number of detectable proteins. Furthermore, plant tissues present an even greater limitation since they contain a high concentration of Rubisco, which is the most abundant protein in leaves [45]. This makes the identification of low-abundant proteins difficult by not selecting their ions for MS^2^. Methods for Rubisco removal have been developed but they result in the removal of similar proteins by lack of antibody specificity, or coprecipitation [46].

The poor protein annotation of non-model plants is an obstacle to protein identification. The use of protein function and interaction tools are good strategies to deal with the lack of functional information since they can highlight annotated interactors and suggest protein groups of overrepresented functions. We implemented this approach to better characterize the three different levels of response to light-induced heat stress obtained in this study.

Proteins with higher expression in the Burned sample following pattern 1 expression were mostly histones, peroxidases, sulfotransferases, and uncharacterized proteins with roles in response to stimulus, carbohydrate metabolic processes, and others. Consistent with a stress response, proteins with a role in biotic stimulus, hormone (cytokinin and abscisic acid), chemical, and other organisms were present in the sample. Redox signals have been reported to be involved in high light acclimation through the electron transport chain, variations in carbohydrate and nutrient status, and hormone levels [47].

**Fig. 8:**
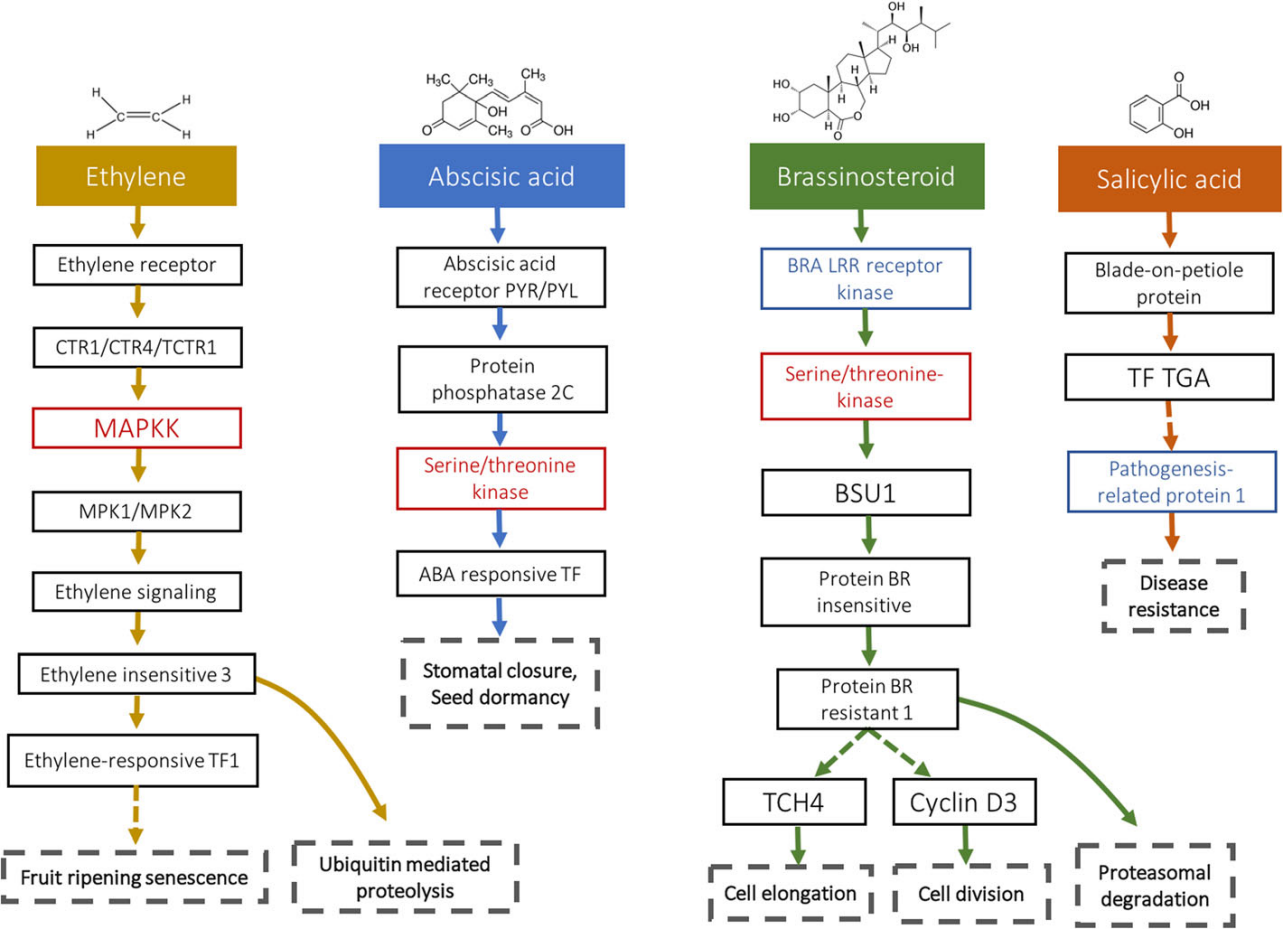
Simplified pathways of the hormones ethylene, abscisic acid, brassinosteroid, and salicylic acid hormones. Proteins found to be more abundant in the Limit and Burned are marked with a dashed-line, proteins only found to be more abundant in the Burned sample are marked with a dotted-line. Dashed boxes are the hormone physiological response. TF: transcription factor, BRA: brassinosteroid

Interestingly, eight proteins found to be more abundant on this dataset were related to plant hormone signal transduction pathways in the Burned samples (Fig. 8). The eight proteins were involved in four hormonal pathways: the abscisic acid hormone (through protein SnRK2) which leads to stomatal closure and stress proteins expression activation [39], the ethylene hormone (through SIMKK), leading to fruit-ripening and stress responses [48], the brassinosteroid hormone (through BRI1 and BSK proteins), which has a role in stem elongation, vascular differentiation and stress tolerance [49], and, lastly, the salicylic acid (PR-1), responsible for disease resistance and inducer of the systemic acquired resistance [50]. No protein related to the salicylic acid pathway was found to be differentially abundant in the Limit sample. This differential expression could be related to the accumulation of heat shock proteins (HSPs) in heat stressed plants, as the salicylic acid has been reported to increase expression of Hsp70/Hsc70 in a dose and time-dependent manner [51]. The hormone cytokinin regulates cell division and maintenance of cellular redox, and most of the cytokinin-regulated genes are involved in response to light and other stimuli [52]. In *Arabidopsis*, high light has been shown to induce CKX6 expression in roots and cytokinin riboside 5′-monophosphate phosphoribohydrolase (protein K4ASD4 in tomato plants) has been shown to be highly responsive to different stimuli [53]. Brassinosteroid is a steroid hormone involved in cell elongation, vascular differentiation, senescence, and stress responses. Brassinosteroid and abscisic acid have been linked to stress responses to heat, oxidation, cold, and pathogens by inducing a rapid and transient NADPH oxidase-mediated H_2_O_2_ production, triggering abscisic acid biosynthesis, increasing H_2_O_2_ production, and prolonging stress tolerance duration [54]. In tomato plants, H_2_O_2_ has been found to be involved in the crosstalk between ethylene and brassinosteroids during salt stress conditions [55].

The Burned sample also presented a differentially expressed protein previously reported as related to the de-etiolation process (CURL-3) [49]. The Burned sample is characterized by the etiolated leaf zone formation, the expression of the CURL-3 protein suggests that the process of de-etiolation was triggered in the Burned leaf zone after the 10-day period.

The protein-interaction network analysis of the Burned sample showed a unique cluster (cluster 3) composed of proteins only related to high light intensity response, those being a group of 10 chlorophyll a-b binding proteins, and the photosystem II component, PsbS. This cluster is evidence of a strong photodamage by enhancing the photosynthetic antenna synthesis. The photosystem recovers using repair cycles for PSII, which requires the monomerization and migration of the phosphorylated dimeric PSII complexes to non-appressed regions of the thylakoid, where all the necessary components for the repair cycle are enriched [56, 57]. D1, D2, and CP43 proteins are dephosphorylated and the degradation of D1 proteins is carried out by FtsH and Deg proteases. The synthesis and thylakoid insertion of D1 is performed by the SecY translocon and ribosomes, and various auxiliary proteins are responsible for the PSII assembly. The D1 and sometimes, D2, PsbH, and CP43 proteins are replaced in the PSII complex while the other members of the complex are recycled [9, 11]. Furthermore, the singlet oxygen radicals near PSII can cause permanent damage to the D1 protein, which is proportional to the light intensity, while the production of superoxide and hydroxyl radicals near the acceptor side of PSI causes oxidative damage to chloroplast lipids and proteins [6].

The light harvesting chlorophyll a-b proteins (LHC) from photosystem II, a group of proteins reported in this study as part of the Burned sample differentially abundant proteins, have been found to have stabilization roles for the PSII supercomplexes structure and increase grana formation through enhancing van der Waals force amongst adjacent thylakoid membranes, and, lastly, in the excitation balance between PSII and PSI [58]. It has been shown that the relative quantity of antenna proteins decrease together with the functional antenna size during high light stress, but LHCII monomers increase during plant acclimation [59].

In our dataset, the antenna complex proteins (LHC) appeared to be downregulated in the Burned sample, along with the reaction center proteins of photosystem II CP43 and CP47, D1, D2, and PSI iron-sulfur center. However, in the same sample, PsbR and PsbS, PsbH, and Psb28 proteins were found to be upregulated. While Psb28 protein has a role in PSII repair along with the CP43-lacking monomer, especially under high-temperature conditions [60], still, the exact mechanism remains unknown. In cyanobacteria, Psb28 has been seen to bind to CP47, and to be involved in the synthesis of chlorophyll and apoproteins of chlorophyll-binding proteins CP47 and PsaA/PsaB [61]. Therefore, the group of upregulated proteins could have similar functions with the aid of the repair and de novo synthesis of PSII complex proteins.

The mRNA levels of PsbS, showed a trend of upregulation in the Burned sample and no change compared to the control in the Limit. Psb28 had no significant change in the Burned sample, when compared to the control, and a statistically significant change (upregulation) in the Limit. In PsbH, the mRNA levels were low in Limit (statistically significant) and Burned. While PsbR presented the same trends, with low levels of mRNA in Burned and Limit samples.

The comparison between the mRNA and protein levels of the four proteins (PsbH, PsbR, PsbS and Psb28), showed a good correlation for PsbS. The lower correlation on Psb28, PsbH and PsbR may indicate a regulation control at the transcriptional level.

## Conclusion

The importance of understanding plants defense from abiotic and biotic stresses relies on the development of strategies to grow plants in adverse conditions. We reported proteins involved in two different levels of light intensity, with expressions that directly respond to the light intensity increase. The identification of differentially abundant proteins during either only the photodamaged/de-etiolation condition or only the medium intensity light in a very localized leaf tissue response. The study of these proteins with a direct response to the light intensity variation is interesting because their genes can be potentially used as light-regulated genetic circuits for biotechnology purposes, as the production of biomolecules of commercial value. We observed the possible exclusive involvement of the salicylic acid hormone in the photodamaged tissue, and we reported on the correlation between protein and mRNA levels of PsbH, PsbR, PsbS and Psb28. Proteins involved in the biosynthesis of salicylic acid were found in the Burned sample, however, the elucidation of the role of salicylic acid role in photodamage should be explored. In this study, uncharacterized proteins were effectively mapped to functions by utilizing the database of proteins interactions. However, a functional genomics study of uncharacterized proteins reported in the differently light-stressed samples would help to characterize the unknown protein roles.

## Additional files

Additional file 1: Spreadsheet (excel file) containing the abundance (ratios) of all proteins identified in all samples. Please refer to Methods for details on the data acquisition, sample preparation and database match. The “Raw Data” tab is a list of all proteins matched to the *Solanum lycopersicum* database under 1% FDR. The “Filtered” tab contains the proteins presenting more than 2 unique peptides. The “DiffExp_Burned”, “DiffExp_Limit” and “DiffExp_Regular” tabs contain a list of proteins found differentially expressed in each of the treatments. Proteins in green font color are upregulated, and the ones in blue color are downregulated. (XLSX 2799 kb)

Additional file 2: Spreadsheet (excel file) containing the identity and information of proteins from each Pattern (1–4) determined through the hierarchical clustering analysis. The “Interaction_Regular” tab shows the protein interaction network of the proteins with roles on immune and stress response found differentially expressed in the Regular sample. (XLSX 1181 kb)

## Abbreviations

BRA: Brassinosteroid
CDF: Cumulative distribution function
ER: Endoplasmatic reticulum
FDR: False discovery rate
GO: Gene ontology
HCD: High-energy collisional dissociation
HSPs: Heat shock proteins
LC: Liquid chromatography
LED: Light emitting diodes
LHC: Light harvesting complex
LHCB: Chlorophyll a-b binding proteins
LHCII: Light harvesting complex of photosystem II
MS/MS: Tandem mass spectrometry
NPQ: Non-photochemical quenching
PICB: Protein interaction cluster of the Burned sample
PICL: Protein interaction cluster of the Limit sample
PR: Pathogenesis-related proteins
PSI: Photosystem I
PSII: Photosystem II
RH: Relative humidity
ROS: Reactive oxygen species
TF: Transcription factor
